# Specific features of ex‐obese patients significantly influence the functional cell properties of adipose‐derived stromal cells

**DOI:** 10.1111/jcmm.17471

**Published:** 2022-07-11

**Authors:** Deborah Schmitz, Jan W. Robering, Volker Weisbach, Andreas Arkudas, Ingo Ludolph, Raymund E. Horch, Anja M. Boos, Annika Kengelbach‐Weigand

**Affiliations:** ^1^ Laboratory for Tissue‐Engineering and Regenerative Medicine Department of Plastic and Hand Surgery University Hospital of Erlangen Friedrich‐Alexander‐Universität Erlangen‐Nürnberg Erlangen Germany; ^2^ Department of Plastic Surgery, Hand Surgery, Burns Center University Hospital RWTH Aachen University University Hospital Aachen Germany; ^3^ Department of Transfusion Medicine and Hemostaseology University Hospital of Erlangen Friedrich‐Alexander‐Universität Erlangen‐Nürnberg Erlangen Germany

**Keywords:** adipose‐derived stromal cells, bariatric surgery, body mass index, cell function, regenerative medicine

## Abstract

Adipose‐derived stromal cells (ADSC) are increasingly used in clinical applications due to their regenerative capabilities. However, ADSC therapies show variable results. This study analysed the effects of specific factors of ex‐obese patients on ADSC functions. ADSC were harvested from abdominal tissues (*N* = 20) after massive weight loss. Patients were grouped according to age, sex, current and maximum body mass index (BMI), BMI difference, weight loss method, smoking and infection at the surgical site. ADSC surface markers, viability, migration, transmigration, sprouting, differentiation potential, cytokine secretion, telomere length and mtDNA copy number were analysed. All ADSC expressed CD73, CD90, CD105, while functional properties differed significantly among patients. A high BMI difference due to massive weight loss was negatively correlated with ADSC proliferation, migration and transmigration, while age, sex or weight loss method had a smaller effect. ADSC from female and younger donors and individuals after weight loss by increase of exercise and diet change had a higher activity. Telomere length, mtDNA copy number, differentiation potential and the secretome did not correlate with patient factors or cell function. Therefore, we suggest that factors such as age, sex, increase of exercise and especially weight loss should be considered for patient selection and planning of regenerative therapies.

## 1 INTRODUCTION

**FIGURE 1:**
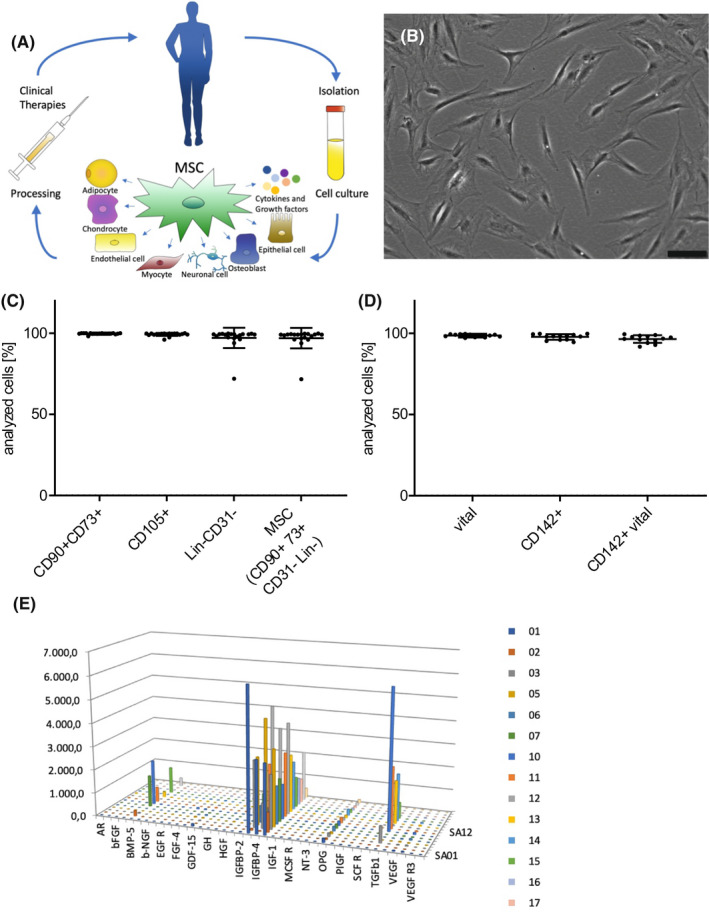
(A) Adipose tissue is an abundant source of stem cells. The so‐called adipose‐derived stromal cells (ADSC) can be harvested from patients in large numbers by minimal invasive surgery. They can be cultivated over several passages and cryopreserved. The secretion of growth factors and cytokines and their ability to differentiate into multiple lineages makes them a promising source for regenerative therapies. ADSC can be used in tissue engineering and clinical applications. (B) Spindle‐shaped, plastic adherent ADSC of patient 14 from passage 1. Scale bar 100 μm. (C) Flow cytometric analysis of isolated ADSC (*N* = 20): positive stem cell markers: CD90, CD73, and CD105; negative markers: Lin (CD34, CD11b, CD19, CD45, HLA‐DR) and CD31. (D) Flow cytometric analysis of isolated ADSC (*N* = 14): 7AAD stained vital cells and CD142 positive cells. (E): Selection of secreted cytokines and growth factors of isolated ADSC (*N* = 17) from different patients (displayed in colour)

In recent years, the transplantation of autologous mesenchymal stromal cells (MSC) has played an increasingly important role in multiple regenerative therapies, including the treatment of various diseases and tissue injuries.^1^ MSC are stromal progenitor cells with proliferation potential and the capability for multilineage differentiation as well as immunomodulation and considered to be a promising treatment modality for tissue regeneration and repair (Figure 1).

MSC are located in adult tissue such as the bone marrow, peripheral blood or adipose tissue, as well as in neonatal tissues such as the placenta and the umbilical cord. Traditionally, MSC have primarily been harvested from bone marrow, which is still a common source. However, in addition to being a painful procedure, isolation can cause infections and rather low cell yields.^2^ While MSC are well investigated and used for intravascular infusions since decades, for example in patients with leukaemia, recently further sources of MSC gained researcher's interest.^3^ Subcutaneous adipose tissue is a favourable source for stem cells, as it is easily accessible and so‐called adipose‐derived stromal cells (ADSC) can be harvested by minimal invasive surgery in high numbers.^4^, ^5^ Like bone‐marrow‐derived MSC, ADSC are multipotent and described to differentiate into various cell lineages, such as adipogenic, chondrogenic, osteogenic, myogenic, cardiomyogenic, neurogenic and angiogenic cells.^6^, ^7^, ^8^, ^9^, ^10^, ^11^, ^12^ ADSC possess immunomodulating properties and secrete various growth factors and cytokines that support tissue regeneration and neovascularization.^13^, ^14^, ^15^, ^16^ Therefore, ADSC are promising not only for tissue engineering^5^ but also for the treatment of chronic wounds and other inflammatory diseases^17^, ^18^, ^19^, ^20^ (Figure 1A).

Since Zuk et al. published a harvesting protocol for ADSC from adipose tissue in 2001, they have become the focus of scientific interest in the field of stem cell research.^21^ In 2016, Naderi et al. concluded from a range of publications, that the multilineage potential of ADSC makes them an optimal cell type for use in tissue engineering and clinical applications.^5^ However, some studies have described variable outcomes of ADSC applications in clinical trials.^22^, ^23^ Differences in harvesting and processing techniques have been well investigated but cannot explain the varying effects of ADSC therapies.^24^, ^25^ Consequently, several studies focused on the influence of patient‐related factors leading to rather controversial results. While some authors could show a correlation between patient‐related factors and cell yield, proliferation and differentiation potential, others could not.^25^ While there are already studies focusing on the secretome of the ADSC and in part on patient‐related factors interfering with the secretion, the influence on ADSC functional properties, such as migratory activity, invasiveness and cell‐matrix interactions, has not been sufficiently investigated.^26^ A better understanding of these parameters is of the utmost importance if these cells are to be used for applications in regenerative medicine. Additionally, the knowledge of beneficial and non‐beneficial properties of cell donors could enable the usage of allogenic ADSC from donors with these beneficial characteristics and simplify the selection of patients.

Therefore, the aim of this study was not only to gain information about cell functions such as viability, migration, transmigration, sprouting and differentiation potential as well as potential differences among ADSC of different patients, but also to analyse the effects of patient‐related factors such as age, sex and BMI on functional cell properties crucial for the application of ADSC in regenerative medicine. A more comprehensive understanding of parameters affecting cell properties could provide important information for patient selection, planning of stem cell treatments and the implementation of regenerative therapies.

## 2 MATERIALS AND METHODS

### 2.1 Patient Cohort

ADSC were isolated between 2017 and 2018 from the subcutaneous abdominal adipose tissue of 20 individuals. Human tissue collection was approved by the Ethics Committee of the Friedrich‐Alexander‐Universität Erlangen‐Nürnberg, Germany (ethics number 264_13B). The patients were surveyed regarding their lifestyle and weight loss history through a questionnaire. An overview of the patient‐specific criteria is presented in Table 1.

**TABLE 1:** Overview of the patient cohort

Sex		
Male	*n* = 5	Patient: 2, 7–9, 19
Female	*n* = 15	Patient: 1, 3–6, 10–18, 20
Age (years)		
Total	43.3 ± 10.9	
≥ 45	*n* = 10 (49.0 ± 7.8)	Patient: 1–3, 9, 11, 13–17
< 45	*n* = 10 (30.0 ± 3.2)	Patient: 4–8, 10, 12, 18–20
BMI		
BMI at timepoint of ADSC isolation	29.8 ± 5.5	
BMI difference	21.1 ± 9.0	
Maximum BMI	50.9 ± 12.5	
Bariatric surgery (gastric sleeve/stapling)	*n* = 12	
Method of weight loss		
Only bariatric surgery	*n* = 4	Patient: 2, 3, 11, 15
Bariatric surgery + diet	*n* = 4	Patient: 6, 13, 14, 17
Bariatric surgery + diet + exercise	*n* = 3	Patient: 12, 18, 19
Diet + exercise	*n* = 6	Patient: 5, 8–10, 16, 20
Not classified	*n* = 3	
Smoking		
Smokers	*n* = 10	Patient: 4, 5, 7, 13–16, 18–20
Non‐smokers	*n* = 10	Patient: 1–3, 6, 8–12, 17
Diabetes mellitus		
Yes	*n* = 0	
No	*n* = 20	
Infection at surgical site before abdominoplasty and isolation of ADSC		
Yes	*n* = 7	Patient: 1, 2, 5, 6, 10, 12, 20
No	*n* = 13	Patient: 3, 4, 7–9, 11, 13–19
Hypertension		
Yes	*n* = 2	Patient: 1, 2, 9
No	*n* = 18	Patient: 3–8, 10–20
Hypothyroidism		
Yes	*n* = 6	Patient: 1, 6, 12, 16, 17, 20
No	*n* = 14	Patient: 2–5, 7–11, 13–15, 18, 19

### 2.2 Isolation and cultivation of adipose‐derived stromal cells

Adipose tissue from post bariatric surgeries was immediately processed under sterile conditions with approximately 40 ml adipose tissue per T75‐flask. The subcutaneous adipose tissue was separated from the skin, cut into standardized pieces smaller than 2 × 2 × 2 mm and washed with phosphate buffered saline (PBS) (Sigma‐Aldrich). Tissue pieces were digested with 1 mg/mL collagenase II (Biochrom GmbH) on an orbital shaker at 37°C for approximately 90 min. Subsequently, the suspension was centrifuged at 400 × g for 10 min, and the supernatant was then aspirated. The pellet was resuspended in minimum essential medium alpha (MEM α) (Thermo Fisher Scientific, Waltham) and filtered (100 μm). Then, the cell suspension was centrifuged (400 × g, 10 min), resuspended in MEM α supplemented with 10% foetal bovine serum (FBS) (FBS superior, Biochrom GmbH) and 1% penicillin/streptomycin (Sigma‐Aldrich) and filtered (70 μm). Cells were counted and seeded into T75‐flasks (mean 4.2 × 10^6^ cells/ml) and incubated at 37°C in 5% CO_2_. Antibiotics were omitted after 48 h, and the medium was changed every 2–3 days. At 90–95% confluency, cells were split and cryopreserved at passage 1 using Accutase (Sigma‐Aldrich) for the detachment and a freezing medium containing MEM α (80%), FBS (10%) and DMSO (Dimethyl Sulfoxide) (Sigma‐Aldrich) (10%). The WST‐8, migration, transmigration and sprouting assay were performed twice in independent assays with cells in passage 2 and 3, with three technical replicates per passage. The differentiation assays were conducted in passage 7 with three technical replicates. For the immunophenotyping by flow cytometry and the measurement of the telomere length and the mtDNA copy number by qPCR cells were used in passage 2, with three technical replicates per passage for the qPCR.

### 2.3 Viability assay

The WST‐8 assay was used for measuring the cell viability. ADSC (2 × 10^3^) were seeded in 96‐well plates in 100 μl of MEM α supplemented with 10% FBS and incubated at 37 °C. After 24 and 72 h, 10 μl of CCVK‐I/WST‐8 solution (Colorimetric Cell Viability Kit I, PromoCell GmbH), was added to each well and incubated for 2 h protected from light. Then, the absorbance was measured at 450 and 600 nm (background) with a microplate reader, and the percentage increase from 24 to 72 h in absorbance was calculated.

### 2.4 Migration assay

To assess ADSC migration, 5 × 10^4^ ADSC were seeded in 100 μl of MEM α supplemented with 10% FBS in 96‐well plates prepared with Oris Cell Seeding Stoppers (Platypus technologies) to create a central cell‐free zone. After 4.5 h, the stoppers were removed, and the cells were incubated for another 48 h at 37°C in 5% CO_2_. Then, the wells were washed with PBS, and the cells were fixed with 4% phosphate‐buffered formaldehyde (Carl Roth GmbH & Co. KG) and stained with 4′,6‐diamidino‐2‐phenylindol (DAPI; 1 μg/ml, 10 min, Life Technologies). Microscopic images were taken at time point 0 h and after DAPI staining (Olympus IX83, cellSens Software, Olympus Corporation). Images at 0 h showing the central cell‐free zone and at 48 h showing stained cell nuclei were merged to count the migrated cells in the region of interest (ROI) (Olympus IX83, cellSens Software). The brightness and the contrast of the images were increased for better visibility.

### 2.5 Transmigration assay

For the transmigration assay, polyethylene terephthalate (PET) membrane transwells (ThinCert, Greiner Bio‐One GmbH) with an 8.0 μm pore size were placed in a 24‐well plate. Then, 5 × 10^4^ ADSC per transwell were seeded in MEM α, and the lower chamber was filled with MEM α supplemented with 10% FBS to stimulate transmigration. After 6 h of incubation at 37°C in 5% CO_2_, the transwells were washed with PBS, fixed with ice‐cold methanol (10 min), stained with DAPI (1 μg/ml, 10 min) and carefully wiped with a cotton swab. Four microscopic images were taken per well, and the stained nuclei were counted (Olympus IX83, cellSens Software). The brightness and the contrast of images were increased for better visibility.

### 2.6 Sprouting assay

For the sprouting assay cells were resuspended in MEM α supplemented with 10% FBS and 0.24% methylcellulose (Sigma‐Aldrich). Then, spheroids of 1,000 ADSC were prepared using the hanging drop (25 μl) technique. After 48 h, the spheroids were carefully washed with PBS and centrifuged at 300 × g for 4 min before being resuspended in MEM α supplemented with 5% FBS and 50 IU/ml thrombin (Tisseel, Baxter International). Subsequently, 0.25% fibrin gels were prepared by mixing the cell suspension in an equal volume of fibrinogen. After a 12‐h incubation at 37°C in 5% CO_2_, images were taken (Olympus IX63). For three spheroids for each experiment, the sprouts were manually counted.

### 2.7 Differentiation assays

For adipogenic differentiation, cells were cultivated in preadipocyte growth medium (PGM) containing Preadipocyte Basal Medium (Lonza) with 10% FBS and 1% penicillin/streptomycin. When confluency was reached, 20,000 ADSC per well were seeded in 24‐well‐plates. Adipogenic differentiation was induced after 5 days by culturing cells in PGM supplemented with 1 μl/ml insulin (Sigma‐Aldrich), 1 μl/ml dexamethasone (sigma), 20 μl/ml 3‐isobutyl‐1‐methylxanthine (SERVA Electrophoresis GmbH) and 4 μl/ml indomethacine (Sigma‐Aldrich). Adipogenic differentiation was assessed after 21 days using Oil red O staining. For osteogenic differentiation, ADSC were cultivated in MEM alpha with 10% FBS. When confluency was reached, 100,000 cells were seeded in 6‐well‐plates. Osteogenic differentiation was induced after 5 days with DMEM LG (Sigma‐Aldrich) with 0.1 μM/ml dexamethasone, 10 mM/ml β‐glycerophosphate (Sigma‐Aldrich), 50 μM/ml ascorbic acid (Sigma‐Aldrich), 10% FBS and 1% penicillin/streptomycin. After 21 days osteogenic differentiation was assessed using alizarin red staining. Media changes for adipogenic and osteogenic differentiation were conducted every 2–3 days.

From both differentiations, images were taken (Olympus IX63) and positive stained areas measured using Fiji (ImageJ). Due to technical issues, samples from patient 1, 3 and 6 could not be analysed for adipogenic differentiation and from patient 1, 3, 6, 7, 10, 13 and 19 for osteogenic differentiation.

### 2.8 Flow cytometry

ADSC at passage 2 were used for flow cytometry to investigate surface marker expression for immunophenotyping (BD FACSVerse, BD Biosciences). The following monoclonal antibodies were used simultaneously according to the manufacturer's specifications: APC mouse anti‐human CD73 (clone AD2), FITC mouse anti‐human CD90 (clone 5E10) and PerCP‐Cy5.5 mouse anti‐human CD105 (clone 266) as positive markers; a PE hMSC negative cocktail, including APC‐Cy7 mouse anti‐human CD31 (clone WM59), CD34 PE (clone 581), CD45 PE (clone HI30), CD11b PE (clone ICRF44), CD19 PE (clone HIB19), HLA‐DR PE (clone G46‐6) (referred to CD34, CD11b, CD19, CD45 and HLA‐DR as Lin‐ markers); and a PE hMSC isotype control negative cocktail, including mIgG1 PE (clone X40) and mIgG2a PE (clone GJ55‐178). Additionally, expression of tissue factor (CD142) was investigated using CD142‐PE (clone HTF‐1) (all from BD sciences). According to the manufacturer's recommendations, daily quality control was performed using Performance QC an Assay Tube Settings setup using BD FACSuite CS&T Research Beads. Between noise and dimly stained cells was discriminated according to the specifications of Wang et al.^27^ To ensure the remaining within the linear range of signal amplification the procedure of Wood et al. and to establish compensation values, the manufacturer's instructions were followed.^28^ The cut‐off value for the positivity was defined by comparison with unstained cells using a histogram, with positive cells being considered to have a signal intensity above the signal intensity of unstained cells. Viability was not measured via flow cytometry, and also the signal‐to‐noise ratio, the linear range and the coefficient of variation were not determined since these parameters were considered to be dispensable for this study. Fluorescence minus one controls were included and reproducibility between the experiments was ensured according to the guidelines for the use of flow cytometry and cell sorting by Cossarizza et al.^29^ Due to technical issues, patient 3 samples could not be analysed. Samples from patient 1, 3, 6, 12, 17 and 19 could not be analysed for the expression of CD142.

### 2.9 Telomere length and mtDNA copy number measurement by qPCR

DNA was isolated using a DNeasy Blood and Tissue kit (Qiagen Science Inc). The average telomere length was measured using an Absolute Human Telomere Length Quantification qPCR Assay kit (ScienCell) and Fast SYBR Green Master Mix (Applied Biosystems). For mtDNA copy number measurements, an Absolute Human Mitochondrial DNA Copy Number Quantification qPCR Assay kit (ScienCell) and Fast SYBR Green Master Mix (Applied Biosystems) were used. Relative telomere length was calculated with the 2^–ΔΔCq^ method. A reference genomic DNA sample with known telomere length and mtDNA copy number served as reference. A single copy reference primer set for a 100 bp‐long region on human chromosome 17 served as reference for data normalization. All kits were used according to the manufacturers' protocols, and samples were tested with three replicates each. Due to technical issues, patient 3 samples could not be analysed.

### 2.10 Quantibody human cytokine array

Conditioned media was prepared by cultivation of ADSC in MEM α supplemented without FCS for 24 h. Afterwards, the media was concentrated and screened for 80 human inflammatory and growth factors (BLC, Eotaxin‐1, Eotaxin‐2, G‐CSF, GM‐CSF, I‐309, ICAM‐1, IFN‐gamma, IL‐1 alpha, IL‐1 beta, IL‐1 ra, IL‐2, IL‐4, IL‐5, IL‐6, IL‐6 sR, IL‐7, IL‐8, IL‐10, IL‐11, IL‐12 p40, IL‐12 p70, IL‐13, IL‐15, IL‐16, IL‐17, MCP‐1, M‐CSF, MIG, MIP‐1 alpha, MIP‐1 beta, MIP‐1 delta, PDGF‐BB, RANTES, TIMP‐1, TIMP‐2, TNF alpha, TNF beta, sTNFRI, sTNFRII Amphiregulin, BDNF, bFGF, BMP‐4, BMP‐5, BMP‐7, b‐NGF, EGF, EGFR, EG‐VEGF, FGF‐4, FGF‐7, GDF‐15, GDNF, Growth Hormone, HB‐EGF, HGF, IGFBP‐1, IGFBP‐2, IGFBP‐3, IGFBP‐4, IGFBP‐6, IGF‐1, Insulin, M‐CSF R, NGF R, NT‐3, NT‐4, Osteoprotegerin, PDGF‐AA, PLGF, SCF, SCF R, TGF alpha, TGF beta 1, TGF beta 3, VEGF‐A, VEGFR2, VEGFR3, VEGF‐D) with a multiplex ELISA antibody array (Human Cytokine Array Q1000, RayBiotech Life Inc.).

### 2.11 Statistics

Statistical analyses were performed with GraphPad Prism (GraphPad Software). The Shapiro–Wilk test was used to test for normal distribution, and the Kruskal–Wallis test with multiple comparisons was used to analyse overall data of the functional assays. For comparisons of age, sex, smoker/nonsmoker and infections/no infections in surgical area before abdominoplasty groups, Welch's test was performed in cases of normally distributed data, while the Mann–Whitney test was used in cases of non‐normally distributed data. To analyse differences between groups of weight loss methods, Welch's anova test was used. In cases of normally distributed data and non‐normally distributed data, Pearson and Spearman correlations were calculated, respectively, to analyse the correlation between BMI at the timepoint of isolation, maximum BMI and BMI difference due to weight loss and the experimental readout. Differences were considered significant at *p ≤ 0.05*. Figures were created with CorelDRAW X6 (Corel Corporation).

## 3 RESULTS

### 3.1 Characterization of isolated ADSC by flow cytometry

From all adipose tissue samples (*N* = 20), ADSC could successfully be isolated and cultivated over several passages. ADSC showed the typical plastic adherence and spindle‐shaped morphology of MSC (Figure 1B). There was no patient‐dependent difference in the number of isolated ADSC. Although not quantified, differences in cell growth rates were observed, leading to variations in culture time until 80–90% confluency was reached. Flow cytometry results showed that 99.77 ± 0.40% of ADSC were positive for the typical markers CD90 and CD73, and 99.13 ± 0.94% of ADSC were positive for CD105. Nearly all ADSC (97.19 ± 6.11%) were negative for the negative markers CD34, CD11b, CD19, CD45, HLA‐DR and CD31 (Figure 1C). All ADSC (96.51 ± 2.27%) were positive for CD142, except for ADSC of patient 18, which showed expression of CD142 on 0.1% of all and no expression of CD142 on vital cells (Figure 1D).

### 3.2 Functional cell assays

**FIGURE 2:**
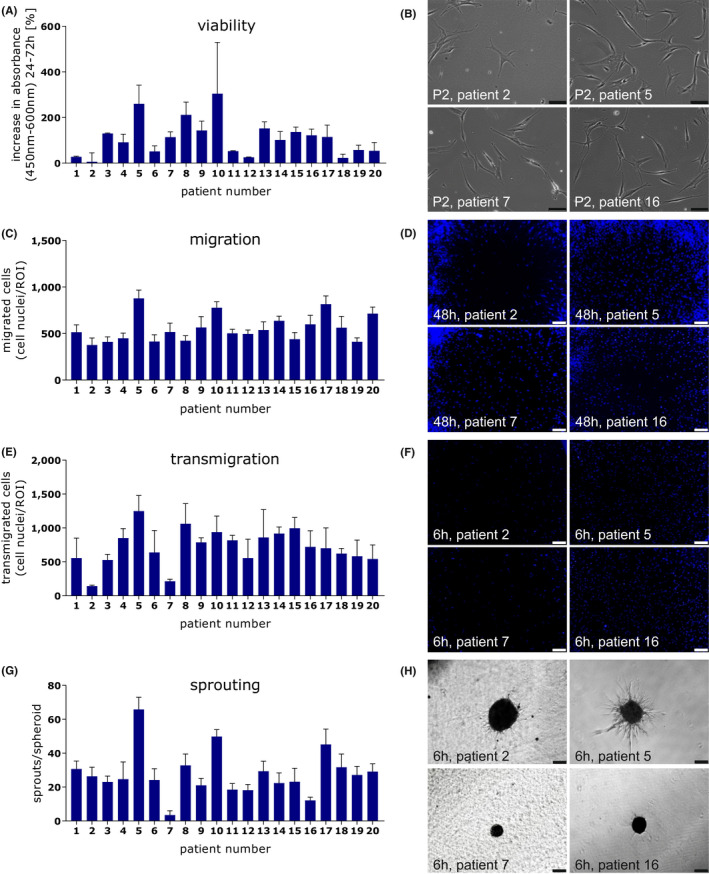
Functional cell assays of isolated ADSC (*N* = 20). (A) WST‐8/cell viability: *Y*‐axis shows the percentage increase in absorbance measured after 24 and 72 h for ADSC from different patients. (B) Representative images of ADSC from four different patients from passage 2. Scale bar 100 μm. (C) Migration: *Y*‐axis shows the number of migrated cells after 48 h per ROI of ADSC from different patients. (D) Representative images of ADSC from four different patients after 48 h, cell nuclei stained with DAPI (blue). Scale bar 200 μm. (E) Transmigration: *Y*‐axis shows the number of transmigrated cells after 6 h per ROI of ADSC from different patients. (F) Representative images of ADSC from four different patients after 6 h, cell nuclei stained with DAPI (blue). Scale bar 200 μm. (G) Sprouting: Y‐axis shows the cell capacity to sprout after 12 h in a 3D‐fibrin‐matrix of ADSC from different patients. (H) Representative images of cell sprouting from ADSC of four different patients taken after 12 h. Scale bar 200 μm

Functional cell characteristics of all ADSC samples were compared through viability, migration, transmigration and sprouting assays. Significant donor‐dependent differences in ADSC proliferation (*p* = 0.0139), migration (*p* < 0.0001), transmigration (*p* < 0.0001) and sprouting (*p* < 0.0001) were observed over time (Figure 2, significances not shown for clarity). While viability was highly variable (Figure 2A, B), migration was less variable (Figure 2C, D). Transmigration was rather similar expect ADSC from patients 2 and 7, showing a considerably lower transmigration potential (Figure 2E, F). The sprouting behaviour of ADSC was highly variable, especially regarding patient 5, ADSC from whom had rather quite high activity and patient 7, ADSC from whom had the lowest activity (Figure 2G, H). Spheroid formation capacity differed as well between the patients, with the ADSC of some patients only forming much smaller spheroids, for example in patient 7, compared with the spheroids of other patients (Figure 2H).

While the ADSC of some patients consistently showed high or low activity in all assays, others displayed more heterogeneous results (e.g. patients 5, 10, 17 reached rather high values in all assays, whereas patients 2 and 7 showed low activity in all assays).

### 3.3 Differentiation assays

**FIGURE 3:**
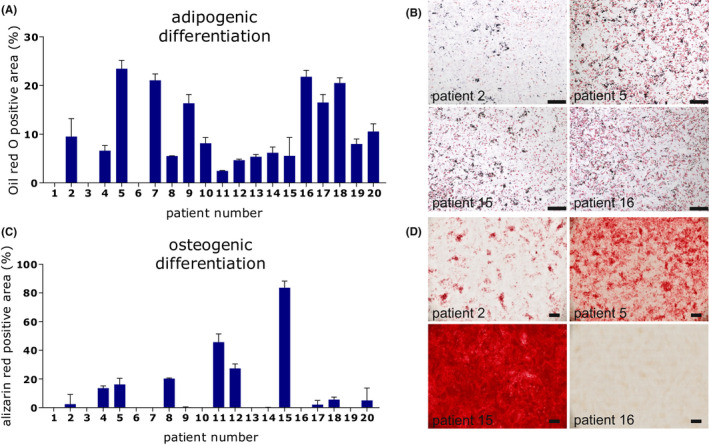
Differentiation assays of isolated ADSC. (A) Adipogenic differentiation: *X*‐axis shows the percentage of positive stained area after 21 days for ADSC from different patients (*N* = 17). (B) Representative images of ADSC from four different patients after 21 days stained with Oil red O. Scale bar 500 μm. (C) Osteogenic differentiation: *X*‐axis shows the percentage of positive stained area after 21 days for ADSC from different patients (*N* = 13). (D) Representative images of ADSC from four different patients after 21 days stained with alizarin red. Scale bar 2 mm

Differentiation potential of ADSC from different patients was compared by adipogenic and osteogenic differentiation assays. Significant donor‐dependent differences in adipogenic (*p* = 0.0001), and osteogenic (*p* = 0.0006) differentiation potential were observed over time (Figure 3, significances not shown for clarity) with high patient‐depending variances. While ADSC of patients 5 and 10 reached comparatively high values for adipogenic differentiation as in the functional assays, osteogenic differentiation potential was rather low. For osteogenic differentiation, ADSC of patient 15 reached outstanding high values, whereas the functional capacity and adipogenic differentiation potential was average.

### 3.4 Patient‐related factors influencing cell function

**FIGURE 4:**
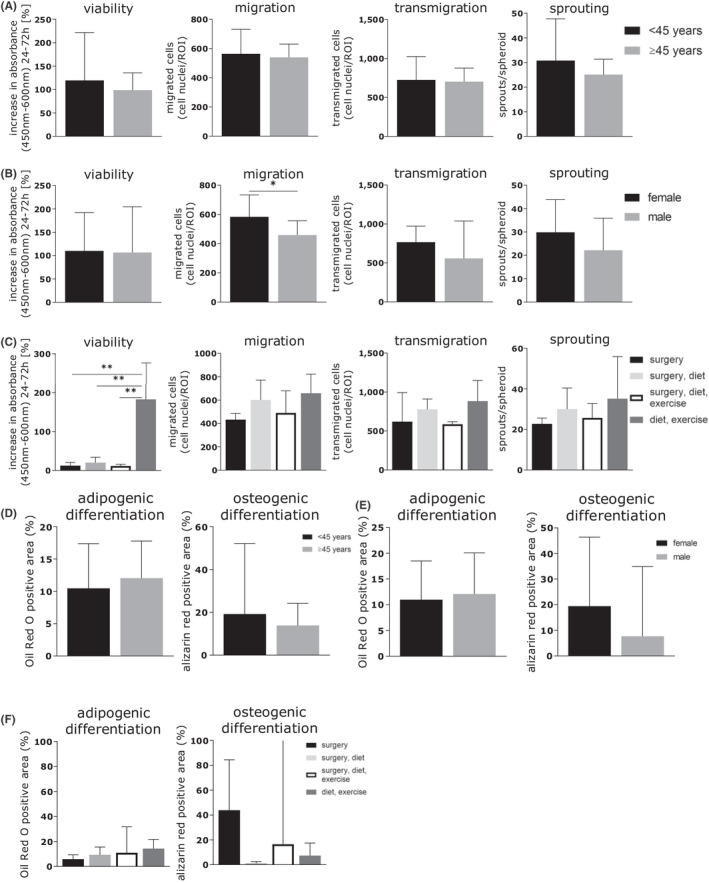
Cell function with regard to patient‐related factors. *Y*‐axes show the viability, migration, transmigration and sprouting capacities. *X*‐axes depict the patient‐related factors: (A) age [< 45 years old (*n* = 10), ≥ 45 years old (*n* = 10)], (B) sex [female donors (*n* = 15), male donors (*n* = 5)] and (C) method of weight loss [bariatric surgery (*n* = 4), bariatric surgery and change of diet (*n* = 4), bariatric surgery, change of diet and increase of exercise (*n* = 3), change of diet and increase of exercise (*n* = 6)]. *p < 0.05, **p < 0.01. Images were generated with GraphPad Prism (Version 8, GraphPad Software, https://www.graphpad.com)

To determine the effects of patient‐related factors on cell function, patients were grouped with respect to age, sex, smoking, infections in the surgical area, BMI and method of weight loss. Additionally, the influence of further comorbidities such as hypertension, hypothyroidism and the medication at timepoint of isolation was analysed. ADSC of patients ≥ 45 years old (*n* = 10) showed lower activity in all four functional assays as well as in the osteogenic differentiation assay than those of patients < 45 years old (*n* = 10) (not significant) (Figure 4A and D). ADSC of female donors (*n* = 15) had a significantly (*p =* 0.0332) higher migration potential and slightly higher viability, transmigration and sprouting potential compared with those of male donors (*n* = 5) (Figure 4B). Furthermore, ADSC of female donors had a higher osteogenic differentiation potential while ADSC of male donors presented a higher adipogenic differentiation potential (not significant) (Figure 4E). Comparing the methods of weight loss [bariatric surgery (*n* = 4); bariatric surgery and change of diet (*n* = 4); bariatric surgery, change of diet, increase of exercise (*n* = 3); diet and increase of exercise (*n* = 6), others not assigned to any of the groups (*n* = 3)], ADSC of patients who combined a change of diet and an increase of physical activity to lose weight showed a significantly higher increase in viability over time than those from patients of the other three groups, including patients who underwent bariatric surgery (surgery *p =* 0.0028, surgery and diet *p =* 0.0041, surgery, diet and increase of exercise *p =* 0.0055). This group also reached higher values in ADSC migration, transmigration, sprouting and adipogenic differentiation although the results were not significant (Figure 4C and F). The factor smoking or infection in the surgical area before abdominoplasty, as well as the other considered comorbidities (hypertension, hypothyroidism) and the medication at timepoint of isolation did not correlate with measured cell function (data not shown).

### 3.5 Influence of BMI on cell function

**FIGURE 5:**
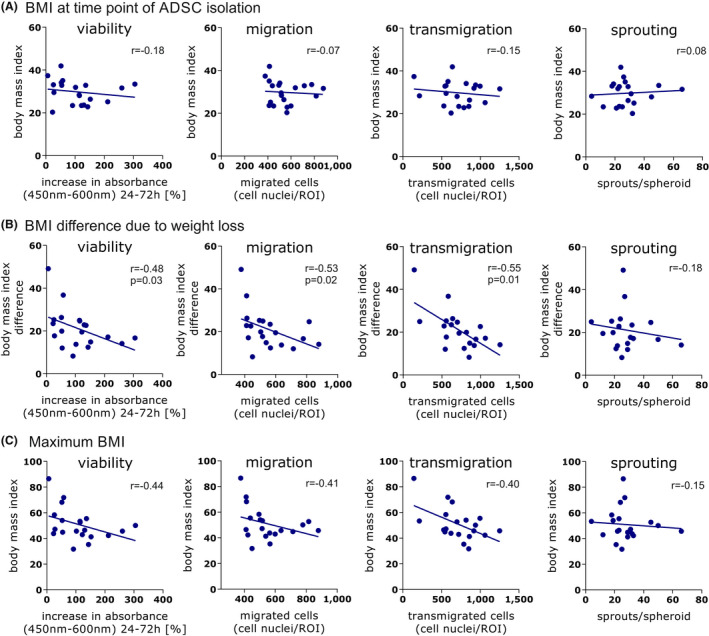
Influence of BMI on functional cell assays. *X*‐axis shows the viability, migration, transmigration and sprouting of ADSC. *Y*‐axis depicts: (A) BMI at the time point of ADSC isolation showing no significant correlation, (B) BMI difference due to weight loss with a significant negative correlation between BMI difference and viability, migration and transmigration and (C) maximum BMI that patients had in the past showing no significant correlation. Images were generated with GraphPad Prism (Version 8, GraphPad Software, https://www.graphpad.com)

Since ADSC were isolated from patients who lost a significant amount of weight, it was possible to investigate the influence of the BMI and BMI change on ADSC function. The BMI at the time point of ADSC isolation showed no correlation with ADSC viability, migration, transmigration and sprouting (Figure 5A). However, the BMI difference was significantly and negatively correlated with the proliferative (*p =* 0.0305), migratory (*p =* 0.0153) and transmigratory (*p =* 0.0119) potential of the ADSC (Figure 5B). ADSC from patients with the highest weight loss leading to a high BMI difference showed a lower activity compared with those from patients with a lower BMI difference. The maximum BMI that patients had in the past showed a moderate negative but not significant correlation with the viability, migration and transmigration of ADSC (Figure 5C). There was no correlation between BMI at time point of ADSC isolation, maximum BMI in patients past nor BMI difference and differentiation potential. Neither a correlation between CD142 expression and BMI could be shown.

### 3.6 Telomere length and mtDNA copy number

**FIGURE 6:**
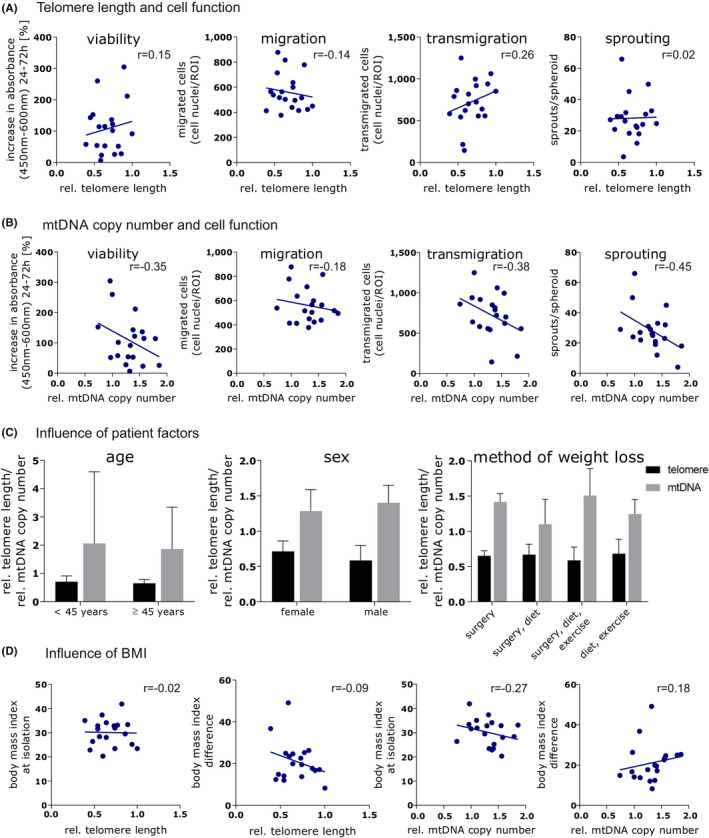
Quantification of telomere length and mtDNA copy number with respect to functional ADSC activity. (A) *Y*‐axes show the viability, migration, transmigration and sprouting capacities. *X*‐axes depict the relative telomere length. (B) *Y*‐axes show the viability, migration, transmigration and sprouting capacities. *X*‐axes depict the relative mtDNA copy number. (C) *Y*‐axis shows the relative telomere length and relative mtDNA copy number. *X*‐axes depict the patient‐related factors: age [<45 years old (*n* = 10), ≥45 years old (*n* = 9)], sex [female donors (*n* = 14), male donors (*n* = 5)] and method of weight loss [bariatric surgery (*n* = 4), increase of exercise and change of diet (*n* = 6), bariatric surgery, change of diet and increase of exercise (*n* = 3), change of diet and increase of exercise (*n* = 4)). (D) *X*‐axes show the relative telomere length and relative mtDNA copy number. Y‐axes depict BMI at the time point of ADSC isolation and BMI difference due to weight loss. Images were generated with GraphPad Prism (Version 8, GraphPad Software, https://www.graphpad.com)

The relative telomere length and the relative mtDNA copy number and their effects on functional ADSC activity of all donors was determined using quantitative real‐time PCR. The relative telomere length and the relative mtDNA copy number were notably different among ADSC, although there was no detectable dependence on the investigated patient‐related factors. Considering age (≥45 years *n* = 9, <45 years *n* = 10) and sex (female *n* = 14, male *n* = 5) as well as the method of weight loss, no significant difference between the groups was observed regarding relative telomere length or relative mtDNA copy number (Figure 6C). BMI at the time point of isolation, BMI difference due to weight loss and the maximum BMI did not correlate significantly with telomere length or relative mtDNA copy number (Figure 6D). In addition, telomere length and mtDNA copy number did not correlate with the viability migration, transmigration or sprouting activity of ADSC (Figure 6A and B). Furthermore, no correlations between smoking and infections in the surgical area as well as other comorbidities and medication and telomere length or mtDNA copy number were observed.

### 3.7 Cytokine secretion

The secretion of inflammatory factors and growth factors was analysed using a multiplex ELISA array. Although we noticed differences in the secretion level among different patients for specific factors, there was no patient or group of patients with constant higher secretion. Additionally, we could not determine a correlation between the investigated patient‐related factors or the observed cell functions and the secretion levels (Figure 1E).

## 4 DISCUSSION

ADSC can be isolated from adipose tissue in large number by minimal invasive surgery. In various experimental and clinical studies, ADSC have been shown to have promise for use in regenerative therapies.^5^ However, notable heterogeneity of ADSC from different donors has been observed, leading to variable outcomes of clinical treatments.^22^, ^23^ For example, long‐term volume retention after autologous fat grafting often varies between patients.^30^, ^31^ Recently, Hurley et al. compared 16 studies on the application of ADSC in osteoarthritis, and although all had positive outcomes, the results were highly varied regarding satisfaction and complication rates.^32^ Also, heterogenity of functional surface marker expression is described. For example, tissue factor (CD142) expression varied among ADSC of different donors, leading to thrombotic events by intravascular application and therefore requires detailed investigation.^33^, ^34^

In the present study, we isolated ADSC from 20 different patients during abdominoplasty after massive weight loss and analysed the effects of patient‐related factors on ADSC functional properties. Differences in ADSC functional activities that are likely of great importance when using ADSC in regenerative medicine, such as viability, migratory and sprouting behaviour as well as differentiation potential were compared with reference to patient‐related factors. ADSC could successfully be isolated from all tissue samples, were plastic adherent and showed a typical spindle‐shaped morphology over several passages. Independent of patient‐specific factors, all cell isolates expressed the surface markers CD105, CD73 and CD90 and were negative for CD45, CD34, CD11b, CD19 and HLA‐DR, meeting the ISCT (International Society for cellular therapy) criteria for identifying MSC.^35^ Further, ADSC of all patients expressed tissue factor (CD142) except for ADSC of patient 18 without expression of CD142 on vital cells. Interestingly, significant differences in the behaviour of ADSC from different donors were observed. While ADSC from some patients showed a consistently high level of activity in all assays (e.g., ADSC of patient 5), others showed rather low activity (e.g., ADSC of patient 2 or 7). The differences among ADSC harvested and processed in the same way led to the assumption that patient‐related factors have a significant impact on the investigated cell characteristics. To analyse these factors with a potential impact on ADSC, the patients were categorized with respect to sex, age, smoking, infection in surgical area, BMI and method of weight loss.

In our analysis, no correlation with sex was observed for viability, transmigration, sprouting activity and differentiation potential of the ADSC, except for migration. Similarly, in the literature, sex is not regarded as an influencing factor on ADSC yield or proliferation.^36^, ^37^, ^38^ Since the only correlation between sex and ADSC characteristics was that the migratory capacity of ADSC from female donors was significantly higher that of ADSC from male donors, we conclude that sex only has a minor effect on ADSC function.

In contrast, in many studies, age is described as a potential influencing factor on the proliferative and migratory potential of ADSC.^39^, ^40^, ^41^, ^42^, ^43^ For example, ADSC of older patients were shown to have a decreased differentiation potential and a lower cell yield than those from younger individuals.^39^, ^40^, ^43^, ^44^, ^45^ However, other authors could not detect any such correlation.^36^, ^46^, ^47^, ^48^ Faustini et al. did not observe any correlation between donor age and cell yield, whereas Choudhery et al. concluded that an advanced age has a negative influence on stem cell function, which could decrease the success of stem cell therapies.^36^, ^40^ In our study, age led to a small but not significant difference in ADSC activity. ADSC from patients <45 years old had a slightly higher activity in all functional assays and a higher osteogenic differentiation potential than those from patients ≥45 years old. However, the low difference between the mean age of both groups (<45 years old: 34 ± 5.34 years old and ≥ 45 years old: 53 ± 4.41 years old) should be considered as well as the absence of very young and elderly patients.

The BMI difference due to weight loss proved to be the strongest influencing factor investigated, with a significant negative correlation observed for the proliferative, migratory and transmigratory potential of ADSC, leading to the assumption that the ADSC of donors who lost more weight showed lower overall activity. These results reflect in part the results of a study by Mitterberger et al., who investigated the influence of bariatric surgery and caloric restriction on ADSC function. The authors observed a decrease in the adipogenic differentiation potential of ADSC from formerly obese patients, whereas the replicative lifespan of these ADSC was increased compared with those from obese and normal weight patients. These results led the authors to conclude that weight loss due to calorie restriction causes a ‘reprogramming’ of ADSC.^49^ Likewise, MacLean et al. described the influence of caloric restriction and weight loss on the gene expression of adipocytes, observing an altered metabolic profile and ADSC function.^50^ Also, Svensson et al. conclude from a range of publications that weight loss induces changes in human adipose tissue gene expression. For example, Clements et al. could show that weight loss was associated with a decrease of proinflammatory factors and an increase of anti‐inflammatory factors.^51^, ^52^ Proinflammatory factors, secreted by cells in the adipose tissue, for example ADSC, are known to promote cell functions such as proliferation and migration. For instance, this correlation was shown for the effect of TNF alpha on MSC by Boecker et al.^53^ The decrease in inflammation due to massive weight loss could possibly explain the lower activity in ADSC of formerly obese patients. On the contrary, ADSC‐conditioned medium was found to promote fibroblast proliferation and migration and thereby support wound healing, restore the inflammatory balance and support lymphangiogenesis.^54^, ^55^, ^56^ Although we investigated the secretome of the ADSC and noticed patient‐depending significant differences for the secretion levels of selected proteins, we could not determine one specific factor explaining the shown functional differences. Additionally, there was no direct correlation between BMI or BMI differences due to weight loss and the analysed cytokines and growth factors. Therefore, we conclude that the interaction between patient‐depending factors, inflammation, caloric restriction and ADSC function is based on complex processes and requires further studies with a larger number of patients to be fully understood. Future studies focusing on cellular differences due to the weight loss and their impact on regeneration for instance in an in vivo animal model or an analysis of the RNA profile will help to confirm our conclusion.

In contrast to the BMI difference, BMI at the time point of ADSC isolation and the maximum BMI of patients in the past had no significant influence on ADSC function. Although some studies came to the same conclusion and could not show any influence of BMI on ADSC proliferation and yield, others described a negative correlation with ADSC proliferation, migration, differentiation and yield.^37^, ^48^, ^57^, ^58^, ^59^

We further analysed the effect of the method of weight loss on ADSC function. Weight loss due to an increase of physical activity and a change of diet was related to ADSC with high activity and significantly higher proliferation, while the group with bariatric surgery without a lifestyle change showed the lowest activity in all assays, except for osteogenic differentiation. This result could be due to a surgically induced severe nutritional deficit leading to a reduction of ADSC activity. However, it should be considered that these observed differences could also be due to an imbalance within groups. While the group of patients who underwent bariatric surgery and did not change their diet nor increase their level of physical activity had a mean maximum BMI of 60.6 ± 17.7 and a mean BMI difference of 28.6 ± 4.9, the group who increased their level of physical activity and changed their diet had a mean maximum BMI of 43.5 ± 13.7 and a mean BMI difference of 15.3 ± 3.0. Therefore, the effect of the observed influence of BMI difference due to weight loss also needs to be taken into consideration.

In 2016, Legzidina et al. described the donor‐specific senescence of ADSC leading to a decrease in cell function.^60^ Regarding age as a potential influencing factor, conditions promoting the aging of ADSC should also be taken into consideration. Therefore, the previously described telomere shortening due to obesity could possibly lead to cell senescence and decrease cell function, resulting in a compromised clinical outcome.^25^, ^26^, ^44^, ^61^, ^62^ Although distinct differences between the relative telomere length of the investigated ADSC were observed in the present study, patient‐related factors had no significant impact on telomere shortening. Interestingly, donor age was not a significant factor influencing telomere length for our investigated ADSC. Again, this result could be due to the patient cohort having a small difference in mean age between the older and the younger groups and the small age range (26–59 years). Furthermore, telomere length did not correlate with cell function.

Mitochondria are important for cellular energy production and cell death, and their number and function are associated with several aging‐related diseases.^63^ Zhang et al. described the influence of mitochondria on stem cell function and their importance for stem cell activity, which can be affected by mitochondrial stress.^64^ Similarly, a study by Li et al. investigated the adaption of mitochondria to environmental and cellular cues and the influence of differentiation of the number and morphology of mitochondria.^65^ While differences between the mtDNA copy number of the investigated ADSC could be shown, no significant effects of patient‐related factors were observed on mitochondria quantity. In addition, mtDNA copy number did not correlate with cellular function within our assays.

Interactions among patient factors confounding the effects of ADSC need to be taken into consideration as limiting factors of this study as well as the absence of a patient group with normal weight without distinct weight loss in the past.

## 5 CONCLUSION

The results of the present study showed significant patient‐specific differences in ADSC functions, such as viability, migration, transmigration and sprouting. Within this study, we could show that age, sex or method of weight loss only had a small effect an ADSC functions. Other influencing factors, such as smoking, diabetes, infections in the surgical area before abdominoplasty, hypertension, hypothyroidism and patient's medication did not correlate with measured cell function. Similarly, BMI at time point of ADSC isolation showed no correlation whereas surprisingly the strongest influencing factor was the BMI difference due to weight loss, with a significant negative correlation observed for the proliferative, migratory and transmigratory potential of ADSC. These findings indicate that ADSC of healthy donors might not have better cell function and demonstrates the importance of a detailed analysis of influencing factors. However, ADSC functional activities are likely influenced by complex interactions among multiple factors. We suggest that patient factors such as age, sex, increase of exercise and especially previous BMI changes can provide helpful additional information for patient selection, planning of stem cell treatments and the implementation of regenerative therapies.

## AUTHOR CONTRIBUTIONS

**Deborah Schmitz:** Conceptualization (equal); formal analysis (equal); investigation (lead); methodology (equal); project administration (equal); visualization (lead); writing – original draft (lead). **Jan Willem Robering:** Conceptualization (equal); methodology (equal); project administration (equal); supervision (equal); writing – review and editing (equal). **Volker Weisbach:** Investigation (equal). **Andreas Arkudas:** Resources (equal); supervision (equal). **Ingo Ludolph:** Resources (equal); writing – review and editing (equal). **Raymund E. Horch:** Resources (equal); supervision (equal). **Anja Miriam Boos:** Conceptualization (equal); methodology (equal); project administration (equal); supervision (equal); writing – review and editing (equal). **Annika Kengelbach‐Weigand**: Conceptualization (equal); formal analysis (equal); project administration (equal); supervision (equal); writing – review and editing (equal).

## CONFLICT OF INTEREST

The authors declare that they have no conflict of interest.

## ETHICS STATEMENT AND CONSENT STATEMENT

All procedures performed in studies were in accordance with the ethical standards of the institutional and/or national research committee and with the 1964 Helsinki Declaration and its later amendments or comparable ethical standards. Human tissue collection was approved by the Ethics Committee of the Friedrich‐Alexander‐Universität Erlangen‐Nürnberg, Germany (ethics number 264_13B). Informed consent was obtained from all patients for being included in the study.

## Data Availability

The data that support the findings of this study are available from the corresponding author upon reasonable request.
